# NANOMEDICINE: will it offer possibilities to overcome multiple drug resistance in cancer?

**DOI:** 10.1186/s12951-016-0172-2

**Published:** 2016-03-09

**Authors:** Sten Friberg, Andreas M. Nyström

**Affiliations:** Department of Neuroscience, Swedish Medical Nanoscience Center, Karolinska Institutet, Retzius väg 8, 171 77 Stockholm, Sweden; Institute of Environmental Medicine, Karolinska Institutet, Nobels väg 13, 171 77 Stockholm, Sweden

**Keywords:** Cancer chemotherapy, Nanomedicine, Multidrug resistance, Cancer stem cells, Tumor-targeted delivery

## Abstract

This review is written with the purpose to review the current nanomedicine literature and provide an outlook on the developments in utilizing nanoscale drug constructs in treatment of solid cancers as well as in the potential treatment of multi-drug resistant cancers. No specific design principles for this review have been utilized apart from our active choice to avoid results only based on in vitro studies. Few drugs based on nanotechnology have progressed to clinical trials, since most are based only on in vitro experiments which do not give the necessary data for the research to progress towards pre-clinical studies. The area of nanomedicine has indeed spark much attention and holds promise for improved future therapeutics in the treatment of solid cancers. However, despite much investment few targeted therapeutics have successfully progressed to early clinical trials, indicating yet again that the human body is complicated and that much more understanding of the fundamentals of receptor interactions, physics of nanomedical constructs and their circulation in the body is indeed needed. We believe that nanomedical therapeutics can allow for more efficient treatments of resistant cancers, and may well be a cornerstone for RNA based therapeutics in the future given their general need for shielding from the harsh environment in the blood stream.

## Background

Around 50 % of humans who are diagnosed with a malignant tumor will die from their disease. In 2012, the actual figures for 40 European countries were 3.45 million new registered cases of various cancers and 1.75 million deaths from malignancies. The global incidence and mortality rates are similar [1]. Despite advances in diagnosis and treatment, mortality from cancer still remains high. Approximately 90 % of the recurrences of cancers after the primary general therapy (endocrine as well as chemical) are caused by the genes coding for multiple drug resistance (MDR) [2–9].

Many cancer therapies kill the bulk cells of a tumor but fail to cure the patient because they do not eliminate cancer stem cells (CSCs), [8, 10–22] which survive to generate new tumors. Virtually all anticancer therapies in use today are designed to target the primary tumors. However, it is usually not the cells in the primary tumor that threaten the life of the patient; it is the metastatic cell population. In the war against cancer, the CSCs and MDR must be mastered. The attacks on the CSCs can be direct (i.e. against the tumor cells) [2–5, 9, 23–25] or indirect (i.e. against in the microenvironment [26–30]) or intended to disrupt the communication between CSCs and the microenvironment [31–37].

Since its discovery in the early 1960s, nanomedicine has created high expectations, and nanotechnology has been expected to reinforce the current medical armament in clinical oncology. Unfortunately, many of these expectations have not been realized. In this review, we will attempt to analyze the present status of nanooncology. Because some publications on the possibilities for nanomedicine have been overoptimistic and oversimplified, we have devoted ample space to present studies showing the difficulties and obstacles that nanooncology faces.

In this review we will present:

1. A brief outline of future oncologic treatments of generalized solid malignancies (in contrast to hematologic malignancies)
2. The biological background to the phenomena of MDR and CSCs
3. The factors that affect drug distribution in the human body
4. A short introduction to nanotechnology
5. An overview of some of the hurdles to be overcome by nanomedicines
6. Strategies likely to be employed in future general oncologic treatments
7. An inventory of drugs available today for general oncologic treatment
8. Some clinical results utilizing nanotechnology.

## An outline of future oncologic treatment of generalized disease

CSCs in a metastatic niche (i.e. stationary) are likely to be protected by several cellular shields, and to reach the CSCs these shields have to be removed one by one, like peeling an onion. The general steps in combatting CSCs include inhibition of drug-resistance mechanisms, elimination of the protecting bulk tumor cells, instigation of growth in the quiescent CSCs, and re-education (in this context—inducing differentiation) or elimination of the CSCs [38]. These steps are described in more detail at the end of this publication.

## The background to MDR AND CSCs

### Definition of mdr

MDR can be defined as a state of resistance against structurally and/or functionally unrelated drugs. The tumor cell population becomes resistant not only to the drug that is initially applied, but also becomes cross-resistant to unrelated drugs with different mechanisms of action [28]. The resistance might be intrinsic in the cell (primary) or acquired via mutations (secondary) [39]. MDR is a basic cellular survival mechanism, and many of the genes involved are ubiquitous. During evolution, they have been maintained from unicellular eukaryotes through multicellular organisms. Bacteria use these or similar genes to become resistant to antibiotics, [40–44] insects use them to overcome pesticides, [40–42, 44–46] and cancer cells use them to survive the oncologist’s treatments.

### Existance of mdr

There are numerous cellular genes involved in MDR. One of the better known is the ATP binding cassettes (ABC) [47–49], a family of more than 50 genes. Several of them control membrane—bound efflux pumps, capable of expelling molecules against a concentration gradient. Many of them collaborate with four of the major cellular signal chains; *WNT*, *NOTCH*, *HEDGEHOG* (Hgh), and *NANOG* (See below). Under normal conditions these four signal chains code for proteins involved in organ development. In cancer cells however they drive the cell population to proliferation. What may be benefical to a baby may be disastrous to its parents.

*Wnt*: ‘W’ stands for “wingless” in *Drosophila*. If the wnt signaling pathway is defective during embryogenesis in Drosophila, the flies do not develop wings. In humans, overexpression of proteins in this pathway leads to multiple basal cell carcinomas in the skin [50–53].

*NOTCH*: Dysfunction in this developmental signaling pathway results in a notch in the wings of *Drosophila* [53–56]. In humans, a dysregulated *NOTCH* is involved in the malignant progression of numerous cancers (breast, pancreas, lung, renal, and also malignant melanoma and malignant glioblastoma multiforme).

*Hgh*: If *Hgh* is dysfunctional during embryogenesis, the larvae of *Drosophila* develop spikes like a hedgehog. In humans, *Hgh* is usually constitutively active in metastatic niches and drives the malignant cell population to proliferate [57–59].

*NANOG*: The term “*NANOG*” has nothing to do with the Greek word “nano” meaning dwarf. It is derived instead from a Celtic myth “Tir nan og” meaning “ever young”, indicating its role in maintaining CSCs in an embryonically young state. NANOG drives cell proliferation to maintain pluripotency and at the same time blocks differentiation [60–66]. Activation of *NANOG* is also a survival mechanism for cancer cells to resist the immune system. High expression of *NANOG* in biopsies from human tumors is correlated with low differentiation, early metastases, and poor prognosis. The collaboration between *WNT*, *NOTCH,Hgh*, and NANOG, drive the malignant cells to become radio resistant, chemo resistant and immune resistant. Their crosstalk can be deadly for the host.

### Cancer stem cells (CSCs)

Populations of both normal cells and cancer cells contain the following four types of cells: resting stem cells, proliferating cells in transit, terminally differentiated cells (which are non-proliferating), and dying cells (apoptotic). Conventional oncologic treatment is directed against the three last components, which constitute the major part of a macroscopic tumor [67].

Even if that therapy is successful, however, the non-proliferating stem cells still remain, and these can instigate proliferation at a later date causing recurrence of the disease [68]. Because these cells are likely to be resistant to further treatment, they are usually fatal. There is no unanimous definition of CSCs, but the American Association for Cancer Research defines them as follows: “A cell within a tumor that possesses the capacity to self-renew and to cause the heterogeneous lineages of cancer cells that compromise the tumor” (http://www.aacr.org). CSCs are identified by the expression of markers on the cell surfaces, by sphere formation in 3D cultures, and by the ability to form growing tumors when transplanted into experimental animals (xenotransplantation). The other three cellular components in a tumor cell population are not capable of xenotransplantation. The occurrence and characteristics of CSCs have been described in numerous publications [7, 8, 10–14, 17–22, 67, 69–87].

The analysis of cell surface markers on CSCs is performed in vitro, but whether the same markers are also expressed in vivo is not known. Neither is it known if normal stem cells express cell surface markers similar to those on CSCs [10, 16, 19, 88]. There is likely to exist a large overlap in phenotypic characteristics and metabolic regulators between normal stem cells and CSCs, and this makes it extremely difficult to design therapies that selectively affect the malignant cells. This information is of paramount importance; if scientists can construct a therapy directed against the cell surface markers on CSCs, it must be known—without doubt—that similar markers do not exist on normal stem cells. Otherwise, the side effects might be lethal to patients. A further complication is that CSCs are not universal for different types of malignancies. For example, the CSCs in a brain tumor might be very different from those in a cancer emanating from a kidney, and the CSCs in the primary tumor might be different from the CSCs in its metastases. Also, CSC’s can differ from one time point to anther in the same tumor cell population. Moreover, the pluripotent CSCs are phenotypically flexible capable of evading the hosts defense mechanisms [67, 84, 89–91].

### Characteristics of CSCs

CSCs are usually rare, and there can be as few as 1 CSC for every 1,000,000 bulk tumor cells [10, 38]. This means that CSCs can be difficult to locate because they are hidden in small niches and difficult to identify by any of today´s diagnostic methods. In the metastatic niche, the CSCs are:

- Protected by their own offspring (whether these cells are dead or alive)
- Capable of evading apoptosis
- Self-sufficient in growth signals (when the tumor is larger than a critical size)
- Capable of limitless replication and self-renewal. Of the four cellular components in a malignant cell population, only the CSCs have such potential
- Able to evade growth suppressors
- Capable of DNA repair
- In possession of forceful efflux pumps
- Initially avascular, thereby not reachable through the blood at early stages
- Able to sustain angiogenesis (when the tumor is over a certain size)
- Capable of tissue invasion
- Mobile
- Capable of cell fusion
- Able to create various phenotypes
- Quiescent. Therefore, they have no or very low metabolism, similar to hibernating animals or dormant plant seeds. Quiescence or dormancy is a property of several different CSCs [10, 16], not only malignant CSCs.

CSCs do not seem to consist of one particular phenotype, but instead appear to represent a plasticity of interchangeable states and a variety of clones. Depending on the selection pressures—which are often epigenetic—one or two clones become dominating in a Darwinian manner. CSCs present an elusive and moving target, defying the hopes of the patient and the efforts of the clinician.

Paradoxically, the host of the malignant cell population facilitates the maintenance of its own enemy: the CSCs. The microenvironment within the host where the CSCs are located not only supports and protects the CSCs, but it also educates the CSCs and instigates MDR. The tumor microenvironment is dominated by the extracellular matrix (ECM) [2, 87, 89, 92–98].

The ECM surrounds almost all somatic cells in higher organisms. It is not only a supportive scaffold but also a dynamic and complex environment that is able to regulate cell behavior. The ECM plays important roles in embryogenesis, cell regulation, and wound healing. The ECM is made up of collagen, elastin, laminin, polysaccharides, and many other biological macromolecules, and the cellular components of the ECM include fibroblasts, macrophages, leucocytes, etc. In cancer patients, the ECM can become deregulated and disorganized in parts of the body, and these regions can harbor metastatic cells in what is known as a metastatic niche [94, 99, 100]. The ECM can even revert mature cancer bulk cells into pluripotent CSCs. The ECM, CSCs, and MDR form an axis of evil, and their cross-talk can be lethal to the host.

## Distribution of drugs in the human body

The distribution of drugs in the human body is governed mainly by vascular transport, transvascular transport, and interstitial transport through the ECM [101]. Extensive reviews are found in the following publications [102–113].

### Vascular distribution

The vascular supply in a solid malignant tumor is heterogeneous, and regions of hypoxia, acidity, and necrosis are intermixed with areas of good vascular supply [114, 115]. No matter where a drug or nanoparticle (NP) is extravasated in a solid tumor they will have difficulties in reaching all regions of that tumor [62, 116].

### Transvascular distribution (or extravasation)

In order to reach their targets, all therapeutic agents must leave the blood circulation. This is not an easy task because the pores in the normal endothelium are very narrow. Normal vessels have 2 nm gaps in capillaries and up to 6 nm gaps in post-capillary venules. Corresponding pores in the kidneys and the liver are 40–50 nm, and in the spleen they are around 150 nm. The pore size in experimental tumors can vary from 100 to 800 nm. Particles can be entrapped in these fenestrae, creating what is called the enhanced permeation and retention (EPR) effect [117].

### Enhanced permeation and retention effect

The vascular beds in experimental animal models are usually malfunctioning. They are leaky and they allow molecules, and even cells, in the circulation to diffuse into the tumor. The enhanced permeability of the tumor vasculature in combination with lack of adequate lymphatic drainage leads to a prolonged half-life (retention) of the drug in a tumor [117–122]. The EPR effect has been utilized by polymer conjugates, micelles, and other NPs that are usually smaller than 200 nm in diameter [123, 124]. However, there are doubts about the therapeutic usefulness of EPR in humans. First, there is no standard EPR effect; it is a highly heterogeneous phenomenon varying from one type of tumor to another, from the primary tumor to its metastases, from one part of a tumor to another, and even within the same tumor at different times. Second, most solid tumors develop high interstitial pressure ranging from 5 to 40 mmHg depending on the tumor type and size. Compared to the normal pressure of <3 mm Hg, such high pressure can effectively counteract the in-flow of therapeutic agents into the tumor [117–120, 125].

Third, the concept that malignant tumors have leaky and irregular vessels is based on observations in experimental tumors that have been selected for fast growth to save time and money for researchers. This is a general observation for most experimental tumors, whether they are chemically or virally induced. The tumor volume doubling time (TVDT) in many experimental tumors is around 10 days. In humans, however, most malignant tumors are slow growing, often requiring years to reach a size of 1 cm^3^. The TVDTs for most human malignant tumors fall between 100 and 300 days [126]. Meanwhile, their vessels can develop slowly and be in good anatomical order with adequate lymphatic drainage. Thus, in humans the value of EPR for therapy of solid malignant tumors is doubtful [120, 127].

Passive transvascular transport is driven by the concentration gradient of NPs from the efferent vessel toward the environment. This transport is a time-consuming process requiring one to a few hours. NPs have no propulsive force, and wherever they land in a body they arrive there through passive distribution [120, 128].

### Interstitial transport

The third mode of distribution concerns molecules as small as oxygen. The interstitium can be vicious and dense, at times very time-consuming to penetrate [128].

All three steps have a profound influence on the distribution of nanomedicines, and these hurdles will be discussed below under the heading “The long journey”.

## Nanoparticles

**Fig. 1:**
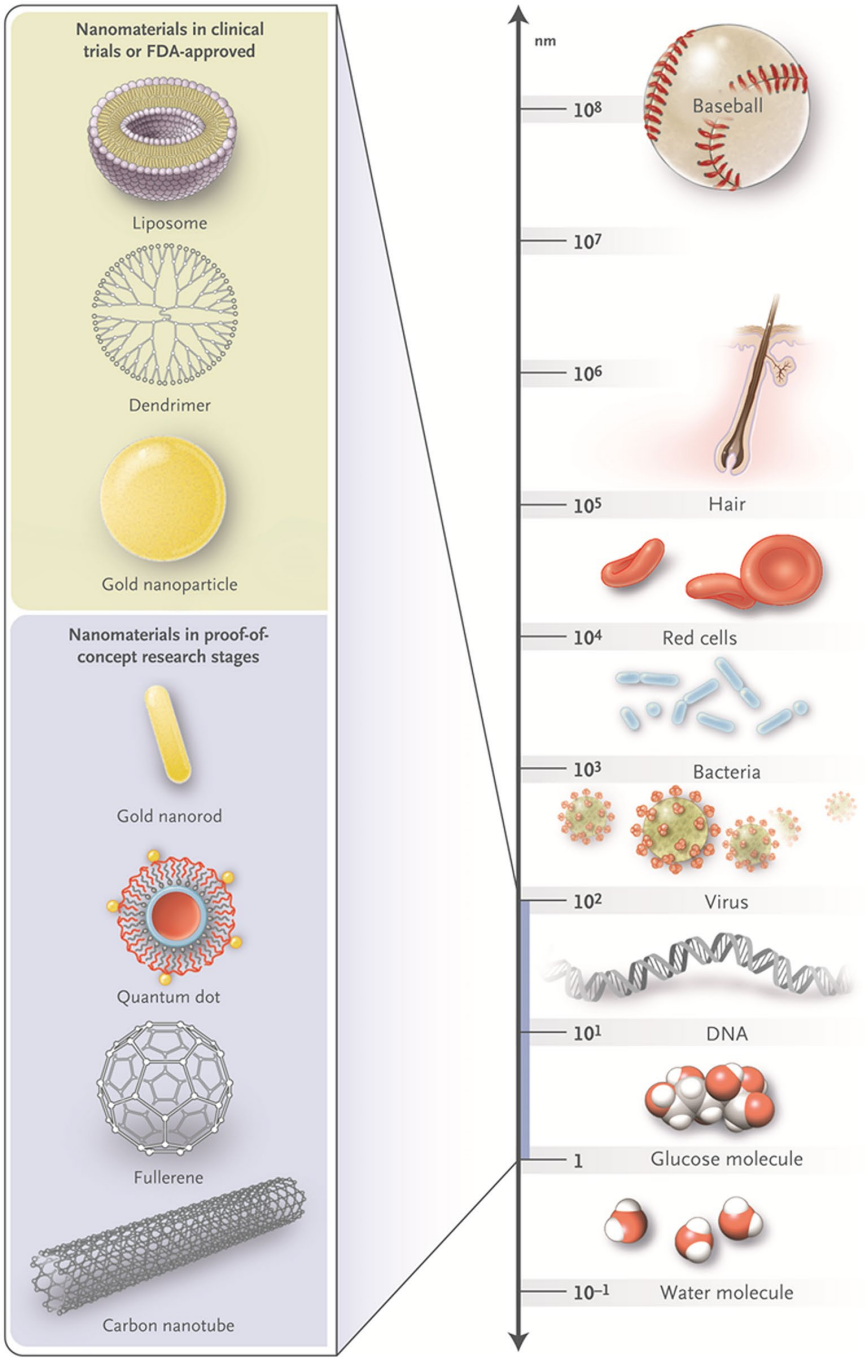
Comparison in sizes between some nanoparticles (*to the left*) and some common objects (*to the right*).with permission from (New England journal of Medicine, Betty YS Kim et al. Nanomedicine 2010; 363: 2437, copyright Massachusetts Medical Society)

Nanos is the Greek word for “dwarf”. Nanotechnology refers to matter with at least one dimension between 1 and 100 nm [129, 130]. One nanometre is 10^−9^ meters, and a sheet of paper is about 100,000 nm thick. In such a world, materials take on different physical, chemical, and biological properties as a result of their small size. NPs are solid particles with a plethora of sizes, compositions, and characteristics [131, 132]. They are usually made of lipids, crystals of metals or silicates, proteins, or polymers. NPs can have several structures, and some of the more common ones are depicted in Fig. 1.

**Fig. 2:**
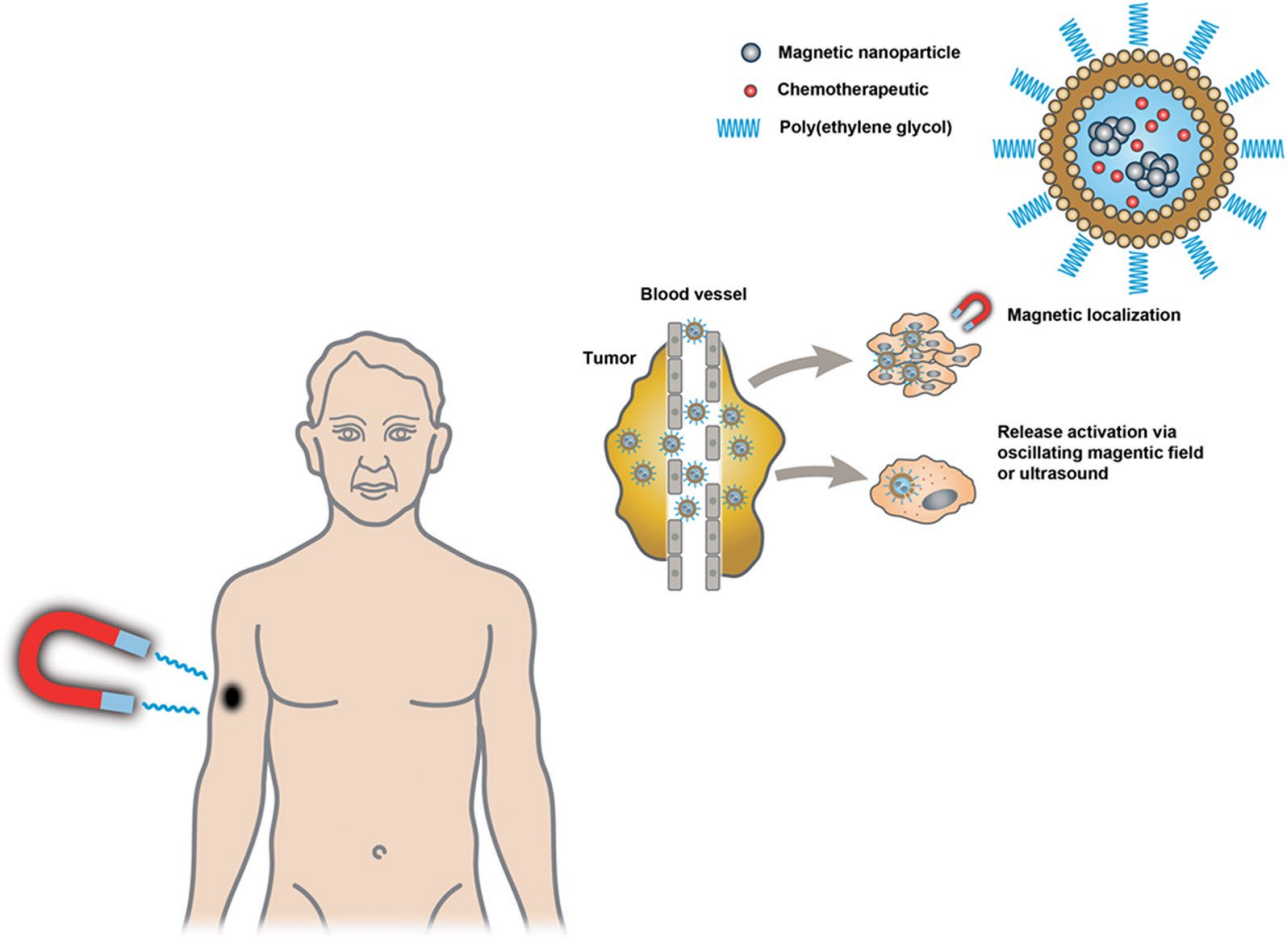
Picture of a person with a tumor on one forearm. The patient is weighing 70 kg. The tumor measures 1 cm in diameter, and it weighs 1 g. If the patient were to be treated with general chemotherapy, then 70,000 g of his body are exposed to the drug, intended for only the malignant 1 g. 99,9999 % of the total number of cells in the patient´s body would—in wanton—be exposed to toxicity [except the cells in the central nervous system (CNS)]. With “targeted therapy” the situation could be completely reversed. The following is what the procedure might be: The drug is loaded into a nanoparticle system (e.g. liposomes). The load also includes magnetic particles (e.g. iron based). The vectors are constructed in such a way that they are dissolved by temperatures exceeding +42 °C. The loaded nanoparticles are given i.v. to the patient. A magnet is attached to the skin near the tumor. In some time (hours), the majority of the nanoparticles will have accumulated in the tumor. Employing microwaves, the temperature in the tumor is then elevated to above +42 °C, causing dissolution of the nanoparticles. The active drug is released, exposing the tumor cells to high concentrations of the drug. Normal cells are spared, and side-effects from the bone-marrow, mucous membranes and the skin are avoided. There is a synergistic effect in utilizing hyperthermia to dissolve the vector: Most malignant tumor cells are more susceptible to elevated temperatures than most normal cells. At +43 °C, the majority of malignant cells are lethally injured, whereas most of the normal cells can recover. It all sounds simple. But in reality, there are numerous obstacles and pitfalls on the road. In this publication, we will point to some of them

NPs can be loaded with drugs, bioactive agents (like genes), or diagnostic tools (like radioactive tracers) [129, 132, 133]. Thus armed, they can serve as vectors inside the body. These vectors can be constructed such that they are activated/dissolved under specific conditions such as acidity, temperature, or light. Such constructs are intended to serve dual purposes of protecting the host from the active agent while it is being transported in the body and protecting the active agent from being excreted by the kidneys, captured by the reticulo-endothelial system (macrophages, antibodies, etc.), or degraded by the host´s normal metabolism. An example of an oncologic therapy based on NP formulations is given in Fig. 2.

There are numerous well written reviews on the topic of nanomedicine and we point the interested reader to the following [28, 81, 132, 134–161].

### Targeting of nanoparticles

So-called targeted delivery is based on functionalization of NPs with tissue-specific ligands such as antibodies, aptamers, [162] peptides, [161, 163, 164] or small molecules [165], and have been described in numerous publications [105, 129, 136, 166–179]. Targets on human cancer cells can be extracellular receptors on the cell surfaces (receptors for transferrin, estrogen, folate [180], etc.) or intracellular receptor (telomere [181], mitochondria [182]). To clarify, in the ligand/receptor interaction, we refer to a ligand as the mobile partner attaching to the non-mobile receptor, like some structure bound to the surface of a cell. In order for any binding between ligand and target to occur, the two participants must be no further apart than a few nm [183]. At greater distances, there is no attraction. This docking is easily achieved in vitro where the medium is of very low viscosity and where there are no mechanical obstacles. In vivo, however, the situation is much more complicated. Repeatedly, it has been shown that targeting of NPs does not increase the number of particles bound to cancer cells in vivo [3, 21, 28, 103, 104, 107, 111, 120, 134, 136, 139, 152, 182, 184–191]. For example, immunoliposomes armed with antibodies against human epidermal receptor (HER) do not bind to cancer cells “overexpressing” HER any more than non-targeted liposomes. The potential and the value of targeting of NPs for clinical oncology have—to date—been overestimated. There are fundamental laws of physics and materials, especially in relation to diffusion, absorption, adherence, and hydrodynamics, that not even NPs can avoid [192]. The situation in vivo is considerably more complex than in vitro making results based on in vitro studies of questionable relevance for situations in vivo. For example “overexpression” of cellular targets is a relative term where the number of structures on the cell surface is compared between a cancer cell and its normal counterpart. The comparison is made under in vitro conditions, but again this might be giving misleading information. First, even if the cancer cells in vitro express 1000 times as many receptors as their normal counterparts do under the same conditions, the relationship might be completely reversed in vivo. Secondly, In vivo, there might be 1,000,000 times as many normal cells expressing the same receptor, all capable of capturing a targeted vector. The results from in vitro analyses have simply underestimated the complexity of the situation. The receptor for transferrin is an illustrative example.

The transferrin receptor (TfR) is exposed on the surface of proliferating cells (recent review in Daniels et al.) [193], and TfR is frequently used as the target in nanooncologic therapy. In the human body, however, there are millions of normal cells expressing very high amounts of TfR. The disease hemochromatosis is a strong and eloquent example of this. Hemochromatosis is a group of diseases characterized by abnormal storage of iron [194]. The metal, which is toxic to cells in high amounts, is stored mainly in the skin, the heart, the pancreas, and the liver–organs where TfRs are abundant [195–197]. These organs are poisoned by the metal. The patient develops diabetes, and the skin is discolored brown. These two clinical features have given the disease the nickname “bronze-diabetes”. The patient usually dies from either heart or liver insufficiency. The clinical picture of hemochromatosis indicates that if a NP is targeted to TfR on the membrane of a cancer cell, and injected intravenously, the NP can be hijacked by TfR expressed in normal organs. Thus the NP is more likely to end up in a normal organ than in a malignant tumor. Aiming at cancer cells with a single surface marker (such as TfR) results in aiming at a single population in a mixture of different cell populations that are constantly changing and moving.

Even if targeting of NPs to cancer cells in vivo can be inefficient, targeting of NPs to receptors on the surfaces of cancer cells has been effective in radiologic detection of cancers in humans [198], and in a few clinical cases targeted NPs have been effective in therapy (see below, Table 3) [108, 109, 113, 198–201].

## The long journey

Until the development of orally formulated nanodrugs, the intravenous route will remain the dominating route for clinical administration of nanooncologic agents. From the entry of the therapeutic NP into the host´s blood circulation, the NP faces a long journey to its intended destination: the cancer cells [27, 107, 127, 186, 202]. During that journey, there are several barriers that need to be overcome. These hurdles are often neglected or disregarded in physiochemical evaluations of the future possibilities of nanotechnology to deliver agents to cancer cells. In this section, we point to a number of different obstacles during this long journey [127, 202].

### Hurdles on the journey

#### Blood

While in the blood circulation, the NPs might have to circulate many times before encountering the tumor. During these circulation passes, they might be captured by macrophages as part of the reticulo-endothelial system. These scavengers are distributed mainly in the liver, the spleen, the lungs, and the bone marrow. The NPs might remain in the blood for several hours, so they must survive numerous capillary meshes (like the lungs and kidneys) where they come into close contact with the host´s blood cells and vascular endothelium.

Thrombocytes—which are abundant in the blood—can capture the NPs through clotting and flocculation [68, 203]. The coagulation system can be activated within seconds to entrap the NPs. The soluble factors in the blood (lipids, proteins, immunoglobulins, enzymes, complement factors, etc.) are likely to alter the surface, the charge, and the size of the NPs by creating a so-called “corona” covering the vectors [149, 204]. This attachment (called “opsonization”) can not only alter the characteristics of the NPs, but also the rate of release of their cargo [205]. Thus even at the beginning of their journey the NPs might begin to lose their cargo (premature delivery). It has been shown, for example, that liposomes loaded with doxorubicin and administered intravenously start to leak 15 min after injection into the bloodstream. After 3 h, only about 10 % of the total administered doxorubicin remains inside the vector [205]. Thus, 90 % of the drug is liberated into the blood where it can cause toxic side effects in the patient. After these hours in circulation, the NPs still might not even have been in contact with the first cancer cell [206]. In this specific example, this effect is similar to the normal administration of doxorubicin, and the slightly reduced release in blood is a strong contributing factor to the lower cardiotoxicity observed for liposomal doxorubicin compared to the free drug. In humans and experimental animals, 95 % of injected NPs end up in normal organs (liver, spleen, lungs), and they are randomly distributed throughout the body by the blood. NPs are typically modified with polymers on their surface to reduce their protein and lipid binding and thus extend their circulation half-life in vivo [207]. This is often achieved with the aid of a hydrophilic polymer such as poly(ethylene glycol). However, the coating of the surface might interfere with the targeting ligands’ ability to bind to their targets because the flexible polymer chains protruding from the NP surface might also block the intended ligand-receptor interaction [208, 209].

From the afferent blood capillary to a tumor cell, the NPs must diffuse through the ECM (interstitial transport). The ECM in a solid malignant tumor is dense, and rigid. It might take several hours for even a small NP (<20 nm) to diffuse a few nm through the ECM. Limited penetration and uneven distribution of chemotherapeutics in solid tumors represent monumental barriers to their efficacy. Even small drug molecules (such as doxorubicin and paclitaxel) remain in close vicinity to the afferent vessels. If poor penetration of small molecules is observed, the penetration of considerably larger NPs is even poorer. Stealth liposomes have been shown to remain adjacent (within 50 nm) to the blood vessels even 2 days after intravenous injection [103, 111, 154–156, 210]. The intratumoral pharmacokinetics/pharmacodynamics are not known for small molecule drugs, let alone the much larger NPs. Small molecules can diffuse through the ECM, but most of them remain localized to regions immediately surrounding the blood vessels, and this leaves large areas of the tumor untouched by the drug [107, 108, 111, 186, 190, 211, 212].

NPs—which are considerably larger than small molecules—can penetrate even less, and then only slowly. It might take several days for a medium-sized NP to travel from the blood vessel to a cancer cell only 200 µm away. It should be noted that most NPs intended for medical use exceed 100 nm in size. Liposomes with a size of 150 nm do not spread beyond 50 µm from the blood vessel [205].

Passive diffusion for an NP up to 10 nm in diameter through a stiff ECM is a time-consuming process, and at times impossible. This delay is in addition to the first two hurdles of the blood and the vascular wall that must be overcome before the ECM is even encountered. Every delay on the long journey can diminish the efficiency of an NP, and improving the intratumoral distribution of drugs/agents is vital for the success of nanooncology to overcome tumors in general and MDR in particular. This is not an easy task. Immunoglobulins (Ig) can serve as an example. IgG molecules (150,000 kDa), which are 1/10 the size of most medical NPs, diffuse 100 nm in 1 h, 1 mm in 2 days, and 1 cm in 7 months. The diffusion speed of considerably larger NPs is not going to be faster [21, 105, 107, 112, 113, 136, 139, 186, 201, 213].

#### Binding site barrier

The first NPs to extravasate create a new barrier to their subsequent followers into the ECM. Moreover, if the NPs manage to immobilize the first cancer cells they encounter, this creates a second barrier [113]. Thus it might not even be possible for NPs to reach the center of a macroscopic tumor.

#### Attachment to the target

When docking occurs between the ligand on the NP and the receptor on the cancer cell, the binding forces are weak and consist of secondary forces [214]. For the interaction to occur, the two participants must be no more than a few nm apart [183]. This is easily achieved in vitro. In vivo, however, the process is much more complicated. In addition, if the vector is targeted with a ligand for a cellular receptor, the ligand must be able to maintain its specificity after its long journey to the cancer cell [109, 134].

#### Endocytosis

A small molecule (<1 nm) can be passively internalized into cancer cells. This is not possible for NPs due to their considerably larger size (see Fig. 1). Thus, they have to enter a cell via endocytosis [199, 215–217]. For a fast-growing cell, this process it a matter of hours. For a slow-growing cell—like a human cancer cell—the whole process can take days [200, 218]. Next, transport through the cytoplasm is a life-threatening passage for an NP, and this part of the long journey is not a question of minutes, but of hours. Once, or if, the NPs reach cancer cells, they face the additional challenge of transport and metabolism inside the cells. Cytoplasmic lysosomes are capable of degrading NPs, and sometimes even rendering drugs inert.

All of today´s cytotoxic drugs intended for clinical use have targets that are located inside the cells. For some gene therapy, the targets are inside yet another barrier, the nuclear membrane.

#### Intracellular transport

This is a complex process which has not been fully elucidated [139, 201]. The diffusion rates of biological molecules inside of a cell depend on several factors. One of the most important factors is the size of the molecule. Small molecules of around 0.5 nm in diameter (like a sugar) diffuse rapidly at around 100 µm^2^ s^−1^. Protein molecules (3–5 nm) diffuse more slowly at around 3–10 µm^2^ s^−1^, whereas larger molecules (like vesicles >10 nm in diameter) move at about 0.1 µm^2^ s^−1^. The time needed for a 10 nm vesicle to traverse a cell 15 µm in diameter amounts to several hours.

#### Release of actives

During its journey from entry into the bloodstream to the moment it reaches the first cancer cell, a major question is whether the vector has been able to retain its drug cargo (see “pre-mature unloading”, page 10). If there is anything remaining of the drug/agent in the vector when arriving at its destination, the release from the carrier can require hours to days depending on the environmental conditions. For example, mesoporous silica NPs release only 30 % of their cargo over 30 h even under optimal conditions [162].

#### The final hurdle

In addition to the hurdles described above, some genes and gene products must also pass through the nuclear membrane. The pores in the nuclear membranes are narrow gates with a mesh of polymers that act as a sieve [99, 219]. Passing these gatekeepers—which some therapeutic genes or gene products must do—is likely to be another time-consuming event. Again, because the NPs have no means of self-propulsion, passing through the pores is a slow process based on passive diffusion driven by a concentration gradient.

From the point of entry (the intravenous syringe) to the target (the cancer cell), there is a considerable distance that a therapeutic agent must travel. For a small NP, this is a very long journey. It is also dangerous. The journey might be a bit shorter in experimental animals than in humans, but it is still a monumental task. The longer this journey takes, the greater the likelihood that the NP will be destroyed or prematurely release its cargo. A rough estimate of the time required for a NP to reach and deliver an active cargo is, at best, around 20 h in the human body. During that journey, how well has that NP been able to maintain its cargo, its specificity, and its integrity?

## Future therapeutic strategies

Therapy against CSCs is likely to require a combination of mathematics, physics, biology, chemistry, and medicine. A short outline of future general oncologic therapy was given in the introduction of this publication. Here, we will give a more detailed sketch of tomorrow´s general oncologic therapy. It is anticipated to be very complex, very personal, and very expensive. CSCs represent the prime target, but these are an elusive and moving target.

The future war on cancer will require multifunctional and multistep sequential therapy. Presented below are some possible steps:

A. Blocking of some of the MDR genes
B. Killing and removing the protecting bulk tumor cells. Just killing is not enough; the dead cells must be removed in order to expose the dormant CSCs
C. Mobilization of the CSCs by instigating them to re-enter the cell cycle
D. Elimination or re-education of the now proliferating CSCs.

### Inventory of agents/drugs available today

Agents/drugs capable of blocking the MDR genes. The scientific community is already in possession of drugs/agents that can block four of the most important signaling pathways involved in MDR in cancer cell populations. Some examples are given in Table 1. However, the potential side effects of blocking these genes are not known. It is also not known in what order they should be blocked, or for how long they must be blocked to allow for the next therapeutic step.

**Table 1:** Examples of drugs/agents capable of affecting some of the MDR genes in human cancer cells

Signal chain	Blocking agents	Clinical status	Ref.
*Wnt*	siRNA Curcumin/Piperin Several other natural products Resveratrol	35 clinical trials	[219–224]
*NOTCH*	siRNA Monoclonal antibodies Peptides Decoys Secretase inhibitors Several natural compounds	15 clinical trials	[56] [54, 225] [225] [226] [53] [227]
*Hgh*	Cyclopamine (alkaloid from plants) Vismodelib Cyclosporin Sulforaphane (Broccoli)	Several clinical trials Clinical trials	[58, 228] [229] [58] [230]
*NANOG*	siRNA Resveratrol	Preclinical	[231] [232]

#### RNA-based therapeutics

Small non-coding RNA sequences can silence gene expression. Several types of RNA exist, but the most well-known is small interfering RNA (siRNA). Several hundred siRNAs have been identified, and each siRNA molecule has the potential to inhibit thousands of genes. Thus the therapeutic possibilities offered by siRNA are enormous [4, 25, 66, 147, 171, 184, 233–255]. siRNA can be synthesized and tailored for specific purposes, and thus these agents are incredibly versatile. However, they have weaknesses. They are unstable, immunogenic, short-lived in plasma, and only function intracellular. The half-life in serum of siRNA are between minutes to an hour, and when endocytosed via nanoparticle uptake the siRNA must be released from the endosome in order to elicit its function in the cytosol. They need a protective vector, which in most applications is an NP. siRNAs have been shown to block MDR genes over the course of 3–7 days in a fast-growing cell population and over the course of several weeks in a slow-growing population [153]. Patil et al. have shown in an in vivo mouse model of drug-resistant tumor, that inhibition of tumor growth can be significant when combining paclitaxel with siRNA for the *MDR1* gene coding for P-gp using a polymeric nanoparticle delivery system that also utilized a biotin as a targeting ligand. The collaboration between nanotechnology and RNA has created a new subspecialty: RNA-Nananomedicine [256].

#### Killing the protective bulk cells

This is the primary goal of today´s oncologic therapy. With the genes for MDR blocked, the bulk cells will hopefully be more susceptible to today´s standard oncologic therapies. Thus, lower doses might be effective, thereby diminishing the side effects in the patient. What is not known is the time required for the body to eliminate the dead cancer cells [257]. This might be a time-consuming process during which time the MDR genes might have begun to function again. The necrotic cells are not likely to be removed by external means; the host´s scavenger cells must do that.

#### Instigating growth in dormant CSCs

Once the protective bulk cells have been removed, the CSCs might have been exposed but still remain quiescent. If so, they must be triggered to re-enter the cell cycle if they are to be treatable. They can be triggered by several already existing agents (review in Wels et al.) [257] thereby becoming susceptible to therapy [87, 258].

Tumor related apoptosis-inducing ligand (TRAIL) is a transmembrane protein of the *TNF* (tumor necrosis factor) gene superfamily that triggers apoptosis in cancer cells, but not in normal cells [215, 247, 259, 260]. Thus it is an ideal candidate for cancer therapy. However, TRAIL lacks clinical applicability because of poor solubility in serum and an unfavorable pharmacokinetic profile. With the aid of nanotechnology, some of these disadvantages can be eliminated.

#### Evaluation of therapeutic effect on CSC’s

Because CSCs are so rare, their elimination cannot be quantified by standard evaluation parameters such as tumor regression rates or retardation of tumor growth rates. Prolonged survival of the experimental animals is regarded as a more relevant parameter [15, 78, 261, 262]. The improvement of survival is interpreted to indicate an effect on the tumors´ CSCs [15, 78, 261–263].

#### Elimination or re-education of the proliferating CSCs

Proliferating CSCs are highly susceptible to conventional chemotherapeutic drugs [6, 149]. Thus, they might be eliminated by low dose chemotherapy [6, 149, 150]. In addition, some of the agents that can block MDR genes (Table 1) can also eliminate CSCs. An alternative to elimination of CSCs is re-education to a differentiated and non-proliferating level. One example given by hematologists: in patients with acute myeloid leukemia (AML), the administration of vitamin A (retinoic acid) prevents a blast crisis [264].

Several of the drugs/agents in Tables 1 and 2 need protection from the host during their transport through the body, and, conversely, the host might need protection from the drug/agent. Sometimes an NP can offer that dual protection. Table 2 lists some of the in vivo experiments where NPs have improved the therapeutic efficacy of a drug or therapeutic agent. In Table 2 we included only publication where the therapeutic effect is measured prolonged survival of the experimental animals.

**Table 2:** Selected examples of investigations where the anti-tumor agent was protected by an NP during transport through the body and where the antineoplastic effect was expressed prolonged survival

Nanoparticle	Targeting ligand	Therapeutic agent	Tumor	Animal	Ref.
Polymer	Biotin	Paclitaxel + tariquidar	“transformed murine”	Mice	[153]
Polymer	None	Dox/curcumin	Four human malignancies	Mice	[265]
Liposome	Peptide	Dox	Human neuroblastoma	Mice	[266]
Chitosan-NP	mirRNA	Paclitaxel	Human ovarian cancer	Mice	[267]
Polymer	Local injector	Dithiazanin	MGB	Rats	[268]
Polymer	Abs, vs ABC G2	Paclitaxel + siRNA	Human breast cancer	Mice	
Polymer	None stated	Dox + Mitomyzin	Human breast cancer	Mice	[269]
Polymer	Photodynamic and chemotherapy	Dox	Human breast cancer	Mice	[270]

*GR* growth reduction, *PS* prolonged survival, *Wt* wild type

**Fig. 3:**
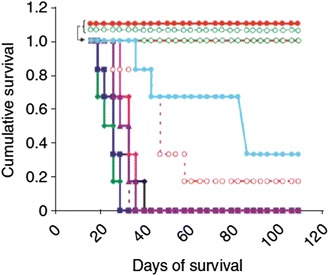
Reversal of multidrug resistance in MDR1-overexpressing aggressive uterine cancer xenografts with complete survival of mice administrated dual sequential treatments. All minicell doses were administrated intravenously in nude mice with 10^9^ minicells per dose. The concentrations of doxorubicin or shRNA administrated per dose in 10^9^ minicells were 1011 copies of shRNA and 0.8 µg doxorubicin. Free doxorubicin was administrated at 150 µg/dose. Kaplan–Meier survival curves for the xenograft study continued for up to 120 days showing complete survival only in the MES-SA/Dx5 mice receiving sequential ^EGFR^Minicell_ss*MDR1*_ and the ^EGFR^Minicells_Dox_ treatment or as expected in mice with the doxorubicin—sensitive MES-SA xenograft treated with ^EGFR^minicells_DOX_. *Black, Control saline*, *green filled*, ^EGFR^Minicell_sh*MDR1*_, *blue filled*, ^EGFR^Minincell_Dox_, *pink filled*, ^CMV^Minicells_sh*MDR1*_ + ^EGFR^Minicell_Dox_, *maroon filled*, ^EGFR^Minicell_sh*Nonsense*_, *purple hollow*, ^EGFR^Minicell_sh*Nonsense*_ + ^EGFR^Minicell_Dox_, *red filled* ^EGFR^Minicell_sh*MDR1*_^EGFR^Minicell_Dox_, *red hollow*, ^EGFR^Minicell_sh*MDR1*_ + free Dox, *green hollow*, ^EGFR^Minicell_Dox_, *Light blue filled*, Free dox

In addition to the examples of possibilities in Table 2 encouraging results have been published by MacDiarmid and her colleagues in Australia [149, 150]. Their series of experiments are worth describing in detail because their results might serve as guidance for the future evolution of nanomedicine. Their experiments used bacterially derived minicells measuring 400 nm in diameter targeted with antibodies against cell surface structures on human cancer cells. Four human cancer cells lines were used: uterus sarcoma, colon cancer, colon adenocarcinoma, and breast cancer. These cells were transplanted into immunodeficient (athymic) nude mice. All four lines were fast growing with TVDTs of 10 days or less, and they were likely to produce pathological vessels thus creating the possibility of an EPR effect. Treatment was carried out in two waves. First, minicells loaded with siRNA capable of blocking the MDR genes *wnt, notch,* and *Hgh* were injected intravenously into the animals. With these genes blocked, the second wave of minicells was administered 2–6 days later. This time the minicells carried cytotoxic drugs, including either irinotecan, or paclitaxel, or doxorubicin, or 5-fluorouracil. The intratumoral presence of the minicells could be observed within 6 h after the injection of the minicells, and 30 % of the drug was found in the tumors compared to only 1 % when the drug was given without the targeted vector [149, 150]. The results were astounding, and growth retardation was observed in all four cancer types and with all four cytotoxic drugs. The doses of the cytotoxic drugs were several thousand folds lower than the doses conventionally used. But even more important, survival of the animals was prolonged indicating that the CSCs in the tumor cell populations were affected. In the group of sequentially treated animals, all six mice survived over the course of the whole observation period of 110 days. In contrast, all of the animals in the control groups had succumbed in less than 50 days (Fig. 3). The critical factor in these experiments is the presence of an EPR effect in the tumors because the size of the minicells precludes transport across normal vessel walls. These results indicate that (1) targeting of NP vectors can be effective, (2) sequential therapy by blocking the genes for MDR prior to anti-neoplastic therapy makes chemotherapy against both cancer bulk cells and CSCs more effective, and (3) survival of the experimental animals is prolonged. As encouraging and exciting as these results might be, however, they still need confirmation from a second independent laboratory.

## Clinical cases where targeted nps are considered to be effective

In spite of all the skepticism, objections, and criticism of NPs—including the hurdles in “The long journey” (see above)—targeting of NPs against cancer cells has been successful in a few clinical cases. We have identified two reports on clinical cases where NPs were employed in the oncologic treatment and where the NPs were traced in some patients. These are summarized in Table 3.

**Table 3:** Reports on clinical cases where therapeutic NPs were traced in the patient

Nanoparticle (NP)	Size of NP (nm)	Ligand/receptor	Drug/agent	Method to trace NP/drug/agent	Ref.
Polymer	70	Protein/TfR	siRNA	PCR	[236]
Liposome	ns	Ab/TfR	p53	p53 via PCR	[271]

*TfR* transferrin receptor, *siRNA* short interfering RNA, *PCR* polymerase chain reaction, *Ab* antibody, *p53* a tumor suppressor gene, *ns* not stated

Davis et al. conducted a phase I study (no effect variable) for treating cutaneous malignant melanoma with siRNA. Biopsies from metastases from malignant melanoma were obtained after completion of the 21 day cycle (drug given on days 1, 3, 8, and 10). The siRNA was designed to suppress the messenger RNA in the malignant cells, and the siRNA in the biopsies was measured with PCR technique. The study demonstrates that siRNA systemically administrated to humans can inhibit a specific gene in a malignant tumor.

Senzer et al. conducted a phase I trial with genetic therapy (a variant of *p53*: SGT 53) against various malignancies. They gave the treatments twice weekly for 5 weeks, and biopsies were taken between 2 and 96 h after the last injection of SGT 53. Tissue samples were taken from cutaneous metastases from three patients with cutaneous malignant melanomas along with normal tissue samples. In all tumor-derived tissue, exogenous ^wt^*p53* was detected, but not in the normal tissue. These results indicate not only the tumor-targeting ability of systemically administered *p53*, but also the specificity for tumor tissue over normal tissue. However, in Senzer et al.’s publication it is not possible to estimate the time required for the NP to travel from the point of administration to the tumor.

## Discussion

The question posed in the title of this article cannot yet be answered with certainty. Tomorrow´s treatments of disseminated cancers face monumental obstacles, but possibilities also remain. The therapy is likely to be personalized, complicated, and expensive. The primary targets—the CSCs—are elusive, evasive, mobile, and changing. New therapeutic strategies and weapons are needed.

An overview of today´s oncologic armament reveals that most of the weapons needed tomorrow already exist today. We are in possession of the drugs needed to block the genes responsible for MDR, we have the agents needed to extinguish the bulk of cancer cells, and we also have the agents needed to instigate growth in dormant cancer cells thereby making them susceptible to therapy. What is missing is knowledge of the chronological order of multistep therapies and the means of directing the active agents to their targets. Nanooncology might offer some solutions to these problems.

The versatility of this young science is very promising and has created high expectations, but several significant obstacles remain before nanomedicine can be considered practical for use in the clinic. One of the main reasons that limit the clinical translation from proof of concept of a novel nanomedicine to clinical phase testing is the matter of multifunctionality. The more complex nanomedicines that one constructs will undoubtable have a limited reproducibility in its manufacturing. An ideal drug delivery system for a MDR treatment may well include both a biological drug cargo and a more standard small molecule, packed in a nanoparticle system with a variation in size (as well as distribution of the cargo). This NP system is then further conjugated (and complicated) with a targeting moiety that allows for tissue targeting. The position and the number of available ligands on the NP surface can then also vary. In concluding such a system with three different components will have a very different composition compared to the “ideal” structure and leads to the issue of which part of this multifactorial system gives rise to the highest efficacy. This aspect is indeed very different from the situation for a small molecular “standard” drug, and this issue is a major limitation. Nanomedicine holds much promise but there are still major areas in both basic and applied research in the area nanotechnology that needs to be explored to solve some of these problems.

Another area of specific concern is the issue of targeting the rare CSC’s. Small molecular therapeutics can indeed diffuse much more efficiently than a 100 nm NP systems and reach both more central parts of a tumor as well as reach more metastatic sites in the body. Such sites are also often less vascularized where small and nanomedicine constructs relying on the EPR effect will not be effective.

## Conclusions

Looking back at the drugs/agents available today (Tables 1, 2, 3 in this publication) that can halt or even cure various generalized cancers, the scientific community is already in possession of the weapons needed. What is missing is aiming and timing. The key is to position the right drug at the right time at the right place and at the right concentration. If this can be achieved, it will represent a major step in treating a wide array of malignancies. In summary the relevance of in vitro based results are questionable still, and tomorrows cancer treatment will need to be multifactorial with different drugs at different time points and perhaps even localized.

## References and notes

Results based on in vitro studies are not included in this list. And we apologize to those researchers whose publications are not listed.
